# Tobacco Use Among 1 310 716 Women of Reproductive age (15–49 Years) in 42 Low- and Middle-Income Countries: Secondary Data Analysis From the 2010-2016 Demographic and Health Surveys

**DOI:** 10.1093/ntr/ntab131

**Published:** 2021-07-22

**Authors:** Radha Shukla, Mona Kanaan, Kamran Siddiqi

**Affiliations:** 1 Department of Health Sciences, University of York, Heslington, York,UK; 2 Hull York Medical School, University of York, York,UK

## Abstract

**Introduction:**

Tobacco use among women, especially during pregnancy is a public health concern. There is a need to understand the diverse nature of their tobacco consumption across the globe.

**Methods:**

We used Demographic and Health Surveys (DHS) data collected between 2010 and 2016 from 42 low- and middle-income countries (LMICs) to estimate the prevalence of smoking, smokeless tobacco, and dual use among pregnant and non-pregnant women of reproductive age (15–49 y). We compared tobacco use between both groups adjusted for age, type of residence, education and combined wealth index, and a subgroup analysis for the South-East Asia Region (SEAR) as the tobacco use in SEAR among women is far more diverse than in other regions primarily due to the popularity of smokeless tobacco use in this region.

**Results:**

Based on the data of 1 310 716 women in 42 LMICs, the prevalence of smoking was 0.69%(95%CI: 0.51–0.90) among pregnant women and 1.09%(95%CI: 0.81–1.42) among non-pregnant women. The prevalence of smokeless tobacco use was 0.56%(95%CI: 0.33–0.84) among pregnant women and 0.78%(95%CI: 0.35–1.37) among non-pregnant women. The relative risk ratios(RRR) for smoking (0.85; 95%CI: 0.67–1.09) and smokeless tobacco use (0.81; 95%CI:0.67–1.00) were not-significantly lower among pregnant women than non-pregnant women and education and wealth index had an inverse relationship with both forms of tobacco. In SEAR, among pregnant women, the prevalence of smoking and smokeless tobacco use was 1.81% and 0.45%, respectively. However, pregnant women were 7%(RRR 1.07; 95%CI:1.02–1.12) more likely to use smokeless tobacco than non-pregnant women.

**Conclusion:**

Despite the added risk of foetal harm during pregnancy, there is no evidence that the tobacco consumption between pregnant and non-pregnant women differ in 42 LMICs. A significantly higher use of smokeless tobacco among pregnant women in SEAR is of particular concern and warrants further investigation.

**Implications:**

Tobacco use among women in low- and middle-income countries (LMICs) is lower than high-income countries (HICs), but this may be because LMICs are earlier in the epidemiological transition of tobacco use. If ignored as a public health issue and the tobacco industry continues to market its products to women, the level of tobacco use may rise as it did in HICs. Also, despite low prevalence rates and with no evidence that these differ among pregnant and non-pregnant women, is concerning as tobacco consumption in any form during pregnancy is associated with poor birth outcomes. This suggests a need for raising awareness about the harms of tobacco use among women in LMICs, especially during pregnancy. There is a need to develop preventive and cessation interventions to decrease tobacco use (smoking and smokeless) among women who are from low socio-economic status and less educated, as they bear the greatest burden of tobacco use.

Tobacco use among women of reproductive age group, especially while pregnant is of particular concern because of the increased risk of fetal growth restriction, preterm births, stillbirths, perinatal deaths, sudden infant death syndrome and placental abnormalities.^1–5^ The evidence related to harmful effects of maternal smoking such as the increased risk of spontaneous abortion, preterm birth, stillbirth, fetal growth restriction, and low birthweight is particularly strong.^6,7^ A cohort study also reports dose-response relation of the number of cigarettes smoked per day during pregnancy and odds of low-birthweight.^7^ However, the evidence on smokeless tobacco use during pregnancy and related adverse outcomes is still emerging.^8^ Relatively few studies have reported an association of low birth weight, stillbirth, small for gestational age, preterm delivery, and anemia to smokeless tobacco use during pregnancy.^9–11^ Despite its widespread use in Asia and Africa, smokeless tobacco is not included in most studies reporting tobacco use among women in the reproductive age.^12^

For low- and middle-income countries (LMICs), standardized prevalence estimates on tobacco use during pregnancy, for both smoked and smokeless tobacco products, are limited.^13^ Lange et al. reported a global prevalence of smoking during pregnancy of 1.7% (95% CI 0.0–4.5).^14^ However, this study did not estimate the prevalence of smokeless or dual use of tobacco during pregnancy. This was addressed by Caleyachetty et al., who reported smoking, smokeless and any tobacco use during pregnancy from 54 LMICs along with regional estimates.^15^ However, these estimates were from the Demographic and Health Surveys (DHS) between January 1, 2001, and December 1, 2012 (DHS phase IV–VI) and many are now outdated.^15^ More DHS waves have concluded (approximately 32 countries with new data), and more data have become available since; updated prevalence estimates are required to report on the contemporary tobacco consumption patterns among pregnant women in LMICs. Due to the additional risk of foetal harms of tobacco use during pregnancy, it is important to report if the prevalence of tobacco use during pregnancy is lower than non-pregnant women of reproductive age. Furthermore, it is also important to assess whether education or socio-economic status determines the use of tobacco among women and if these differ by pregnancy status. Both of these issues have not been addressed by the previous two studies.^14,15^

We estimated the recent prevalence of tobacco use among women of the reproductive age group disaggregated by their pregnancy status and explored its socio-demographic determinants. Our estimates are based on 42 LMICs, with a specific focus on the South East Asia region (SEAR) due to the popularity of smokeless tobacco use in this region.^16,17^

## Methods

We analyzed cross-sectional survey data from the DHS waves six and seven (2010–2016). These are nationally representative surveys conducted systematically in the LMICs using standard methods detailed elsewhere.^18^

The sample was based on a stratified two-stage cluster design: in stage one, census files were used to draw enumeration areas, based on which a sample of households was drawn in stage two. An initial household questionnaire was completed by interviewing one member, who also provided a list of other household members. All consenting women aged 15–49 y were further interviewed to answer the women and child questionnaire. The surveys broadly provided information on maternal and child health variables including socio-demographic characteristics, antenatal care, family planning, child health, nutrition, tobacco use, HIV prevalence and attitudes/beliefs and women empowerment. These were normally conducted over a period of 18–20 mo and were of two types. Standard DHS are conducted about every five years and collect information from a larger sample size (usually between 5000 and 30 000 households). Interim DHS are mainly conducted for monitoring indicators and are conducted in the duration between standard surveys. For the purpose of this study, we used standard DHS data conducted between 2010 and 2016, which reported tobacco use history (including both smoking, smokeless tobacco and dual-use) in 42 countries.

The survey reported tobacco consumption as a binary variable, as current use of various forms of tobacco. Broadly these include cigarettes, pipes, cigars, chewed, snuffed, and country-specific tobacco. Therefore, to calculate the prevalence, a new variable was generated to classify tobacco use. The outcome variable for the analysis was tobacco use categorized into exclusive smoking, exclusive smokeless, dual and no tobacco use (reference category). The covariates were pregnancy status asked as “are you currently pregnant?” and reported as a binary variable (“Yes” or “No or not sure”), age recorded as a continuous value (years completed), area of residence (“urban” or “rural”) and education (“no education,” “primary,” “secondary,” or “higher”), reported as categorical variables. Socioeconomic status is calculated as a combined wealth index for the household based on selected household assets and reported categorically (“poorest,” “poorer,” “middle,” “richer,” and “richest”).^19^

### Statistical Analysis

Data were analyzed in STATA version 15,^20^ using the *Individual Recode* files from each country. Sampling weights were calculated to account for differential probabilities of selection and participation, and to account for the two-stage stratified cluster sampling strategy of the DHS design. From each country’s survey data set, women were categorized as pregnant and non-pregnant. Characteristics of these women based on mean age and proportions for the type of residence, education, and wealth index distribution were calculated for each country.

Prevalence estimates were generated along with 95% confidence intervals for tobacco use among pregnant and non-pregnant women. Furthermore, pooled prevalence estimates of all 42 countries combined with 95% confidence intervals (CI) were computed.

Multinomial regression analysis was performed to assess the determinants of the different types of tobacco use by women and Relative Risk Ratios (RRR) were estimated. The explanatory variables included pregnancy status, type of residence, education, wealth index, and age. For this, we pooled data from all the countries into one data set. The analysis also accounted for clustering based on countries and *p*-values less than 0.05 were considered statistically significant.

A similar analysis was performed for SEAR, which included India, Indonesia, Myanmar, Nepal, and Timor-Leste. Pooled prevalence estimates with 95% confidence intervals (CIs) were computed and multinomial regression analysis (with a similar outcome and explanatory variables) was performed, accounting for clustering based on countries.

## Results

We analyzed data of 80 512 pregnant and 1 230 724 non-pregnant women (these include missing data) from 42 countries. The response rate for tobacco use, both smoked and smokeless, among women in all 42 countries was more than 99%, which summed up to 80 454 pregnant and 1 230 262 non-pregnant women combined from all 42 countries from 2010 to 2016. The characteristics of these women from each country are listed in supplementary tables.

The mean age of women varied across countries ranging from 23.6 y (Nepal) to 28.8 y (Ghana) among pregnant women and 27.4 y (Gambia) to 33.2 y (Pakistan) among non-pregnant women. The proportion of women with no formal education ranged from 0% (pregnant women) and 0.05% (non-pregnant women) in the Kyrgyz Republic to 85.6% and 82.9% in Afghanistan, respectively. Furthermore, nine countries had more than 50% of pregnant women with no formal education, while the same was in eight countries for non-pregnant women.

Prevalence of exclusive smoking was 0.69% (95%CI: 0.51–0.90) among pregnant women and 1.09% (95%CI: 0.81–1.42) among non-pregnant women. Prevalence of exclusive smokeless tobacco use was 0.56% (95%CI: 0.33–0.84) among pregnant women and 0.78% (95%CI: 0.35–1.37) among non-pregnant women across all 42 countries (Tables 1 and 2). Prevalence of dual tobacco use was 0.03% (95%CI: 0.01–0.06) among pregnant women and 0.08% (95%CI: 0.05–0.11) among non-pregnant women. Furthermore, dual tobacco use during pregnancy was zero in 25 of the 42 countries, with the highest of 0.26% (95%CI: 0.003–1.82) in the Kyrgyz Republic. Additionally, a comparison of tobacco use among pregnant and non-pregnant women for smokeless tobacco and smoking, are presented in the supplementary figures.

**Table 1.** Tobacco use Among Pregnant Women

Tobacco use during pregnancy					
Country	Response rate %	Response rate n (weighted)	Exclusive smokeless % (95% CI)	Exclusive smoking % (95% CI)	Dual % (95% CI)
**Afghanistan (2015)**	99.71	6393	1.78 (1.39–2.26)	2.29 (1.77–2.96)	0.25 (0.12–0.52)
**Angola (2016)**	99.88	1362	0.005 (0.001–0.27)	1.05 (0.62–1.79)	0.12 (0.002–0.55)
**Armenia (2016)**	100	174	0	0	0
**Benin (2012)**	100	1556	0.68 (0.33–1.41)	0.21 (0.006–0.66)	0
**Burkina Faso (2010)**	99.73	1725	2.77 (2.01–3.79)	0	0
**Burundi (2016)**	100	1420	1.79 (1.11–2.87)	1.76 (1.11–2.78)	0.25 (0.003–1.75)
**Cambodia (2014)**	99.88	932	1.16 (0.64–2.09)	0.66 (0.25–1.7)	0.39 (0.18–0.88)
**Cameroon (2011)**	99.69	1507	0.009 (0.002–0.39)	0.12 (0.003–0.52	0
**Comoros (2012)**	99.22	348	1.34 (0.29–6.06)	1.34 (0.39–4.5)	0
**Congo (2012)**	99.9	1029	0.75 (0.38–1.48)	0.45 (0.15–1.32)	0.005 (0.001–0.23)
**Cote d’Ivoire (2012)**	99.86	1031	1.03 (0.52–2.02)	0.42 (0.1–2.21)	0
**Dominican Republic (2013)**	100	479	0	1.69 (0.72–3.92)	0
**Ethiopia (2016)**	100	1135	0.004 (0.001–0.13)	1.25 (0.38–4.0)	0.001(0.0001–0.009)
**Gabon (2012)**	99.76	812	0.009 (0.002–0.44)	2.24 (1.0–4.91)	0.008 (0.002–0.33)
**Gambia (2013)**	100	830	0	0	0
**Ghana (2014)**	100	663	0.12 (0.001–0.86)	0.008 (0.001–0.6)	0
**Guatemala (2015)**	100	1427	0	0.11 (0.003–0.36)	0
**Haiti (2012)**	100	837	2.58 (1.46–4.54)	0.88 (0.45–1.71)	0.15 (0.002–1.06)
**Honduras (2012)**	99.92	1213	0	0.71 (0.27–1.83)	0
**India (2016)**	100	31 123	3.21 (2.94–3.5)	0.43 (0.35–0.52)	0.005 (0.002–0.11)
**Indonesia (2012)**	99.96	1949	0.28 (0.12–0.65)	0.73 (0.4–1.35)	0
**Kenya (2014)**	99.97	1943	0.71 (0.46–1.09)	0.51 (0.18–1.44)	0
**Kyrgyz Republic (2012)**	99.93	550	0	0.53 (0.13–2.13)	0.26 (0.003–1.82)
**Lesotho (2014)**	100	284	4.71 (2.55–8.55)	0	0
**Liberia (2013)**	100	765	0.11 (0.002–0.46)	0.13 (0.004–0.4)	0
**Malawi (2016)**	100	1874	0.003 (0.0005–0.22)	0.49 (0.21–1.14)	0.009 (0.002–0.33)
**Mali (2013)**	100	1202	1.16 (0.48–2.78)	0.008 (0.001–0.34)	0
**Mozambique (2011)**	100	1516	0	0.35 (0.11–1.07)	0.43 (0.17–1.07)
**Myanmar (2016)**	99.97	465	0	3.36 (1.95–5.73)	0
**Namibia (2013)**	100	600	0.21 (0.006–0.73)	2.93 (1.73–4.92)	0.3 (0.007–1.19)
**Nepal (2016)**	100	535	0.46 (0.13–1.63)	1.66 (0.68–3.99)	0
**Niger (2012)**	99.86	1588	1.1 (0.66–1.82)	0	0
**Pakistan (2012)**	99.84	1458	1.64 (0.81–3.29)	3.55 (2.38–5.25)	0.21 (0.004–1.11)
**Philippines (2013)**	100	686	0.36 (0.11–1.16)	2.32 (1.4–3.83)	0
**Rwanda (2015)**	100	984	1.04 (0.51–2.08)	0.25 (0.007–0.82)	0.15 (0.003–0.66)
**Sierra Leone (2013)**	99.78	1425	3.45 (2.28–5.17)	2.09 (1.39–3.12)	0.1 (0.001–0.73)
**Tajikistan (2012)**	99.83	732	0	0.11 (0.001–0.81)	0
**Tanzania (2016)**	100	1135	0.26 (0.007–0.85)	0.57 (0.24–1.32)	0
**Timor-Leste (2016)**	100	690	0	4.92 (2.85–8.37)	0
**Togo (2014)**	100	807	0.26 (0.003–1.85)	0.006 (0.0009–0.48)	0
**Uganda (2016)**	100	1843	0.76 (0.45–1.27)	0.74 (0.36–1.53)	0.001 (0.0001–0.007)
**Zambia (2014)**	100	1427	0.82 (0.36–1.88)	0.009 (0.001–0.51)	0
**Total**		**80 454**	**0**.**56 (0**.**33–0**.**84)**	**0**.**69 (0**.**51–0**.**90)**	**0**.**03 (0**.**01 – 0**.**06)**

**Table 2.** Tobacco use Among Non-Pregnant Women

Tobacco use among non-pregnant women					
Country	Response rate%	Response rate n (weighted)	Exclusive smokeless % (95% CI)	Exclusive smoking % (95% CI)	Dual % (95% CI)
**Afghanistan (2015)**	99.65	22 968	2.45 (2.08–2.87)	3.45 (2.93–4.07)	0.26 (0.17–0.4)
**Angola (2016)**	100	13 015	0.11(0.006-0.19)	1.8 (1.49–2.19)	0.09 (0.05-0.1)
**Armenia (2016)**	99.98	5941	0	1.07 (0.76–1.49)	0.21 (0.09–0.51)
**Benin (2012)**	100	15 043	0.61 (0.48–0.78)	0.23 (0.16–0.33)	0.05 (0.02–0.11)
**Burkina Faso (2010)**	99.9	15 342	3.95 (3.44–4.55)	0.08 (0.04–0.15)	0.02 (0.006–0.07)
**Burundi (2016)**	100	15 849	2.66 (2.33–3.04)	1.84 (1.56–2.16)	0.009 (0.005–0.17)
**Cambodia (2014)**	99.98	16 640	3.61 (3.17–4.11)	1.89 (1.48–2.41)	0.55 (0.41–0.74)
**Cameroon (2011)**	99.81	13 887	0.49 (0.33–0.71)	0.29 (0.21–0.39)	0.04 (0.01–0.03)
**Comoros (2012)**	99.73	4965	2.97 (2.21–3.96)	1.59 (1.18–2.15)	0.15 (0.06–0.32)
**Congo (2012)**	99.87	9775	1.55 (1.23–1.94)	0.47 (0.31–0.71)	0.06 (0.04–0.11)
**Cote d’Ivoire (2012)**	99.66	8997	1.23 (0.96–1.58)	0.31 (0.17–0.58)	0.07 (0.01–0.36)
**Dominican Republic (2013)**	99.9	8884	0.04 (0.005–0.29)	4.66 (4.06–5.34)	0.009 (0.001–0.06)
**Ethiopia (2016)**	100	14 548	0.05 (0.02–0.13)	0.52 (0.33–0.84)	0.04 (0.01–0.12)
**Gabon (2012)**	99.69	7585	0.19 (0.008–0.4)	3.02 (2.43–3.76)	0.17 (0.08–0.33)
**Gambia (2013)**	99.77	9381	0.04 (0.01–0.09)	0.19 (0.1–0.34)	0.01 (0.001–0.08)
**Ghana (2014)**	99.97	8730	0.32 (0.2–0.52)	0.07 (0.02–0.23)	0.03 (0.04–0.21)
**Guatemala (2015)**	99.94	24 473	0.01 (0.003–0.04)	1.59 (1.4–1.82)	0.02 (0.006–0.03)
**Haiti (2012)**	99.84	13 429	2.82 (2.34–3.39)	1.88 (1.59–2.22)	0.36 (0.24–0.53)
**Honduras (2012)**	99.94	21 530	0.03 (0.01–0.07)	1.79 (1.54–2.08)	0.006 (0.002–0.02)
**India (2016)**	100	668 563	4.61 (4.48–4.73)	0.76 (0.72–0.81)	0.05 (0.04–0.06)
**Indonesia (2012)**	99.9	43 613	0.31 (0.23–0.42)	2.37 (2.13–2.62)	0.1 (0.06–0.17)
**Kenya (2014)**	99.98	29 130	0.4 (0.32–0.5)	0.2 (0.13–0.31)	0.01 (0.005–0.06)
**Kyrgyz Republic (2012)**	99.94	7652	0.007 (0.001–0.05)	2.88 (2.29–3.62)	0.02 (0.002–0.14)
**Lesotho (2014)**	100	6338	7.6 (6.73–8.57)	0.31 (0.18–0.53)	0.02 (0.006–0.09)
**Liberia (2013)**	99.96	8469	0.5 (0.36–0.71)	0.35 (0.22 -0.57)	0.007 (0.001–0.06)
**Malawi (2016)**	100	22 688	0.11 (0.07–0.18)	0.4 (0.31–0.52)	0.19 (0.12–0.29)
**Mali (2013)**	100	9222	0.96 (0.69–1.33)	0.15 (0.08–0.26)	0.05 (0.01–0.17)
**Mozambique (2011)**	100	12 229	0.14 (0.06–0.31)	0.8 (0.61–1.04)	0.72 (0.54–0.98)
**Myanmar (2016)**	100	12 419	0.17 (0.09 -0.29)	3.63 (3.14–4.19)	0.03 (0.009–0.09)
**Namibia (2013)**	99.91	8567	0.6 (0.46- 0.78)	4.14 (3.6–4.76)	0.24 (0.16–0.38)
**Nepal (2016)**	100	12 327	2.68 (2.32–3.09)	5.29 (4.76–5.88)	0.71 (0.51–0.99)
**Niger (2012)**	99.88	9569	2.49 (1.8–3.43)	0.02 (0.003–0.13)	0.009 (0.001–0.06)
**Pakistan (2012)**	99.84	12 087	2.4 (1.91–3.0)	3.94 (3.19–4.84)	0.12 (0.06–0.24)
**Philippines (2013)**	99.97	15 464	0.38 (0.25–0.57)	5.7 (5.26–6.18)	0.18 (0.12–0.26)
**Rwanda (2015)**	99.94	12 505	1.11 (0.91–1.37)	1.05 (0.85–1.29)	0.07 (0.03–0.14)
**Sierra Leone (2013)**	99.79	15 197	3.63 (3.08–4.27)	4.32 (3.78–4.93)	0.39 (0.27–0.55)
**Tajikistan (2012)**	99.75	8900	0.04 (0.01–0.11)	0.2 (0.1–0.38)	0
**Tanzania (2016)**	100	12 131	0.45 (0.32–0.62)	0.45 (0.27–0.74)	0.02 (0.005–0.08)
**Timor-Leste (2016)**	100	11 917	0.14 (0.08–0.25)	3.97 (3.4–4.63)	0.09 (0.04–0.17)
**Togo (2014)**	99.86	8661	0.57 (0.37–0.89)	0.16 (0.07–0.33)	0
**Uganda (2016)**	100	16 663	0.55 (0.41–0.73)	0.78 (0.64–0.96)	0.04 (0.02–0.08)
**Zambia (2014)**	99.9	14 969	1.1 (0.86–1.41)	0.32 (0.23–0.44)	0.14 (0.07–0.29)
**Total**		**1 230 262**	**0**.**78 (0**.**35–1**.**37)**	**1**.**09 (0**.**81–1**.**42)**	**0**.**08 (0**.**05–0**.**11)**

The number of observations in the regression analysis (all eligible women from 42 countries after excluding missing values, which were less than 0.001%) was 1 310 651 (Table 3). There was no statistically significant difference in exclusive smoking (RRR of 0.85, 95% CI of 0.67–1.09) and smokeless tobacco use (RRR of 0.81, 95% CI of 0.67–1.0) between pregnant and non-pregnant women.

**Table 3.** Multi-Nominal Regression Analysis (All Countries Combined)

Multinomial logistic regression Number of observations = 1 310 651			
Dependent variable: tobacco use	RRR	*p*-value	95 % CI
No tobacco use	Base outcome		
**Exclusive smoking**			
Pregnant (ref = no)			
Yes	0.85	0.217	0.67–1.09
Residence (ref = urban)			
Rural	0.56	<0.001	0.44–0.71
Education (ref = no education)			
Primary	0.87	0.5	0.56–1.32
Secondary	0.68	0.01	0.51–0.91
Higher	0.65	0.13	0.37–1.15
Wealth index (ref = poorest)			
Poorer	0.65	<0.001	0.59–0.71
Middle	0.47	<0.001	0.39–0.57
Richer	0.39	<0.001	0.28–0.48
Richest	0.26	<0.001	0.18–0.39
Age	1.05	<0.001	1.04–1.06
Constant	0.009	<0.001	0.006–0.012
**Exclusive smokeless**			
Pregnant (ref = no)			
Yes	0.81	0.048	0.665–0.998
Residence (ref = urban)			
Rural	0.89	0.51	0.63–1.25
Education (ref = no education)			
Primary	0.72	0.29	0.39–1.32
Secondary	1.07	0.6	0.83–1.39
Higher	0.64	0.03	0.43–0.96
Wealth index (ref = poorest)			
Poorer	1.01	0.88	0.90–1.13
Middle	0.76	<0.001	0.68–0.85
Richer	0.51	<0.001	0.45–0.59
Richest	0.24	<0.001	0.19–0.29
Age	1.06	<0.001	1.05–1.07
Constant	0.13	<0.001	0.005–0.03
**Dual tobacco use**			
Pregnant (ref = no)			
Yes	1.01	0.94	0.80–1.27
Residence (ref = urban)			
Rural	0.72	0.02	0.54–0.96
Education (ref = no education)			
Primary	1.11	0.79	0.53–2.29
Secondary	1.14	0.71	0.56–2.32
Higher	0.53	0.02	0.31–0.89
Wealth index (ref = poorest)			
Poorer	0.73	0.05	0.53–1.0
Middle	0.65	0.07	0.41–1.03
Richer	0.68	0.184	0.38–1.2
Richest	0.39	<0.001	0.24–0.64
Age	1.07	<0.001	1.06–1.08
Constant	0.0003	<0.001	0.0002–0.0005

With respect to the type of residence, women living in rural areas had a lower relative risk of tobacco smoking compared to women living in urban areas (RRR of 0.56, 95% CI of 0.44–0.71). However, there was no statistically significant difference in the use of exclusive smokeless tobacco use between women who live in urban and rural areas (RRR: 0.89, 95% CI of 0.63–1.25).

Women with at least secondary education had a statistically significant reduction in exclusive smoking (RRR of 0.68, 95% CI of 0.51–0.91) when compared to women with no tobacco use, while women with at least higher education had a statistically significant reduction (RRR of 0.64, 95% CI of 0.43–0.96) in smokeless tobacco use compared to women with no formal education (*p*-value 0.03).

The wealth index was also a significant predictor of tobacco use among women and showed an inverse relationship. The relative risk for smoking reduced from 0.65 (95% CI of 0.59–0.71) in the 2^nd^ quintile (poorer) to 0.26 (95% CI of 0.18–0.39) in the highest quantile (richest) when compared to the reference category of the poorest. Similarly, the relative risk of smokeless tobacco use decreased with every quintile increase in wealth index (RRR of 1.01and 95% CI of 0.9–1.13 in the poorer wealth index to RRR of 0.24 and 95% CI of 0.19–0.29 in the richest wealth index). In terms of dual-use, women in the richest quintile of wealth index had a RRR of 0.39 (95% CI of 0.24–0.64) compared to women in the poorest wealth index quintile.

In SEAR countries, during pregnancy, the pooled prevalence of smoking was 1.81% (95% CI of 0.61–3.61) and of smokeless tobacco use was 0.45% (95% CI of 0.002–2.29) (Supplementary Table). Regression analysis (Table 4) which accounts for five countries, had 783 588 observations. Wealth index was a significant predictor for both smoking and smokeless tobacco use, however, education was significant only for smoking. Furthermore, there was no evidence of a statistically significant difference in smoking among pregnant and non-pregnant women (RRR 0.88, 95% CI of 0.73–1.05); in fact, pregnant women were 7% more likely to use smokeless tobacco than non-pregnant women when compared to no tobacco use (RRR 1.07, 95% CI of 1.02–1.12).

**Table 4.** SEAR—Multinomial Regression Analysis

Multinomial logistic regression – SEAR Number of observations = 783 588			
Dependent variable: tobacco use	RRR	*p*-value	95 % CI
No tobacco use	Base Outcome		
**Exclusive smoking**			
Pregnant (ref = no)			
Yes	0.88	0.157	0.73–1.05
Residence (ref = urban)			
Rural	0.62	<0.05	0.44–0.87
Education (ref = no education)			
Primary	1.22	0.19	0.9–1.65
Secondary	0.67	0.001	0.53–0.85
Higher	0.43	<0.001	0.32–0.57
Wealth index (ref = poorest)			
Poorer	0.6	<0.001	0.55–0.65
Middle	0.4	<0.001	0.36–0.45
Richer	0.3	<0.001	0.25–0.37
Richest	0.18	<0.001	0.13–0.25
Age	1.06	<0.001	1.04–1.07
Constant	0.009	<0.001	0.005–0.014
**Exclusive smokeless**			
Pregnant (ref = no)			
Yes	1.07	0.01	1.02–1.12
Residence (ref = urban)			
Rural	0.76	0.09	0.55–1.05
Education (ref = no education)			
Primary	1.07	0.82	0.6–1.89
Secondary	0.98	0.92	0.69–1.39
Higher	0.6	0.02	0.39–0.93
Wealth index (ref = poorest)			
Poorer	1.08	0.2	0.96–1.2
Middle	0.81	0.04	0.67–0.99
Richer	0.55	<0.001	0.41–0.75
Richest	0.28	<0.001	0.18–0.42
Age	1.05	<0.001	1.05–1.06
Constant	0.03	<0.001	0.02–0.04
**Dual tobacco use**			
Pregnant (ref = no)			
Yes	1.1	0.53	0.82–1.48
Residence (ref = urban)			
Rural	0.56	<0.001	0.45–0.77
Education (ref = no education)			
Primary	1.67	0.19	0.77–3.6
Secondary	1.33	0.4	0.68–2.61
Higher	0.51	0.06	0.25–1.03
Wealth index (ref = poorest)			
Poorer	0.87	0.31	0.66–1.14
Middle	0.86	0.27	0.66–1.12
Richer	0.94	0.69	0.7–1.27
Richest	0.56	<0.001	0.44–0.72
Age	1.06	<0.001	1.05–1.07
Constant	0.0005	<0.001	0.0002–0.0011

## Discussion

To the best of our knowledge, this study is the first of its kind providing nationally representative estimates of smoking, smokeless tobacco and dual-use among women in the reproductive age group based on a large sample of over 1.3 million from 42 LMICs. The study also further quantifies how the use of tobacco varies with pregnancy status, level of education, wealth index, age, and residence when compared to women with no tobacco use.

Tobacco smoking during pregnancy has declined over a period of time; we report an overall prevalence of smoking during pregnancy in 42 LMICs as 0.69%, which is lower than previous estimates by Caleyachetty et al. as 1.3% based on 54 LMICs, and by Lange et al. as 1.7%, who reported a global prevalence based on 147 countries.^14,15^ We also report a prevalence of tobacco smoking among non-pregnant women as 1.1%. Based on our estimates, tobacco smoking might have been less prevalent among pregnant women (0.69%) than non-pregnant women (1.09%); indicating the possibility of women trying to avoid smoking during pregnancy. In relative terms, the difference is similar to the difference in smoking prevalence before and during pregnancy derived from the UK National Infant Feeding Survey (~50%).^21^ However, contrary to the UK estimates, ours were not based on the same cohort of women therefore any speculations on women reducing smoking during pregnancy in these 42 LMICs are subject to change with future cohort data. Besides, our estimates were not statistically significant after adjusting for age, type of residence, level of education and wealth index. In the US, the prevalence of smoking in 2016 dropped from 13.5% in adult women^22^ to 7.2% during pregnancy.^23^ A study from Greece reports that 63.4% of pregnant women gave up smoking during pregnancy.^24^ A difference in the prevalence of tobacco use between pregnant and non-pregnant women was not seen in our study. Lange et al reported that women who smoke daily and continue to smoke during pregnancy are high in the regions of Africa and Asia, compared to other regions and thus, this could potentially be due to most women not quitting when becoming pregnant in these regions.^12,14^ However, given the cross-sectional nature of this study, it cannot be stated that some women continue to smoke when they are pregnant, as both groups (pregnant and non-pregnant) consisted of different individuals. However, in our study, the difference did become significant in women who were more educated which could be a proxy for heightened awareness of tobacco-related risks to the fetus.

In SEAR, we found that smokeless tobacco use was more common during pregnancy. This may be due to the previous suggestions that some women might start using smokeless tobacco during pregnancy as a relief for morning sickness, to combat bad taste or watery sensation in the mouth.^25–27^ However, whether these women started smokeless tobacco use during their course of pregnancy or changed its frequency, cannot be confirmed due to the limitations of the DHS questionnaire and further research is warranted to explore this possibility.

Education and combined wealth index are significant predictors of tobacco use across all three categories of tobacco use when compared to no tobacco use among women. Exclusive smoking is less likely among women with at least secondary education while exclusive smokeless tobacco and dual tobacco use are less likely among women with at least higher education. With respect to the wealth index, an inverse relationship is clearly evident; with every increase in the quintile of wealth index, the RRR of tobacco use decreases for both exclusive smoking and smokeless tobacco use among women. This is consistent with previous literature^28–31^ which suggests that tobacco use is more prevalent in the low-socioeconomic population and those in women with no formal education.^32^ A reason for pre-dominance among women from less privileged populations besides lack of awareness might be due to the use of smokeless tobacco in suppressing hunger while performing difficult laborious tasks^30^; besides smokeless tobacco is generally cheap and its use by women is not stigmatized.^33–35^ Therefore, this study strengthens the evidence as to why tobacco cessation services and awareness need to be targeted to women from low socioeconomic status.

### Strengths

Firstly, the study offers the advantage of a large sample in that it is nationally representative and covers 42 countries. Moreover, the questionnaires and method of data collection are uniform allowing for cross-country comparisons. Secondly, the study provides prevalence estimates for all eligible women in the reproductive age group and for pregnant women. As these estimates are from the same survey data set, they allow comparisons within the country of prevalence rates at that specific time point. Lastly, to the best of our knowledge, the study is a first of its kind, which reports regression analysis on such a large sample comparing various categories of tobacco use with socio-demographic variables as predictors.

### Limitations

The study uses data from cross-sectional surveys and as a result, only suggests possible sociodemographic predictors and no causation can be elucidated. All of the collected data is self-reported and thus there is a possibility of under-reporting tobacco use estimates. Also, the survey mainly asks women about their extensive reproductive and maternal history, which could potentially cause hesitancy towards accurate tobacco use reporting. This is contradictory to specific tobacco surveys such as the Global Adult Tobacco Survey (GATS) that only aim to collect tobacco use in detail with trained interviewers. For example, the GATS conducted in India in 2016 reported a 2% prevalence of smoking and 12.8% of smokeless tobacco use among women, compared to our estimates of 0.43% and 3.21% respectively.^36^ However, our observations are consistent with results from previously conducted DHS analysis by Caleyachetty et al.^15^

The prevalence rates may be an underestimate and with no evidence that these differ significantly between pregnant and non-pregnant women, is concerning. This suggests the need for more awareness related to tobacco use during pregnancy in LMICs and offer them the support to quit/reduce tobacco during pregnancy. Similar to high-income countries, where many have developed interventions to reduce smoking during pregnancy, may also help in LMICs.^13^ In general, this warrants the need for tailored smoking cessation advice for pregnant women in LMICs.

Furthermore, based on our estimates, smokeless tobacco use was more common among pregnant women than non-pregnant women in SEAR. This is an important area where further research needs to focus as the literature suggests women starting smokeless tobacco during pregnancy for multiple reasons and later continuing its use due to addiction.^26,37,38^ Along with a further understanding of smokeless tobacco use in pregnancy, it is also equally important to educate and support women from low socioeconomic status and those with low levels of education. In LMICs, various maternal and child health interventions are delivered through community health workers (CHW) and there is some evidence that the preventive interventions delivered by CHW might be effective.^39^ Hence, a potential opportunity is to deliver targeted preventive and cessation services through the local CHW as part of routine maternal and child health programmes. Also, current tobacco control policies primarily focus on smoking and the inclusion of smokeless tobacco in policy formation and implementation is essential to reduce smokeless tobacco use among women of reproductive age.^40^

## Supplementary Material

A Contributorship Form detailing each author’s specific involvement with this content, as well as any supplementary data, are available online at https://academic.oup.com/ntr.

ntab131_suppl_Supplementary_Material

ntab131_suppl_Supplementary_Taxonomy_Form
