# Supplementary material for: Tobacco Use Among 1 310 716 Women of Reproductive age (15–49 Years) in 42 Low- and Middle-Income Countries: Secondary Data Analysis From the 2010-2016 Demographic and Health Surveys
Source: Nicotine Tob Res. 2021 Jul 22;23(12):2019–27. doi: 10.1093/ntr/ntab131 (PMC8849114; doi:10.1093/ntr/ntab131)
Supplement: ntab131_suppl_Supplementary_Material [file ntab131_suppl_Supplementary_Material.docx]

Supplementary Table 1: Characteristics of pregnant women across countries

| **Characteristics of pregnant women (n = 80512)** | | | | | | | | | | | | |
| --- | --- | --- | --- | --- | --- | --- | --- | --- | --- | --- | --- | --- |
| **Country** | **Pregnant women (weighted)** | **Mean age** | **Rural dwellers (%)** | **Highest education level (%)** | | | | **Wealth Index (%)** | | | | |
|  |  |  |  | **No education** | **Primary** | **Secondary** | **Higher** | **Poorest** | **Poorer** | **Middle** | **Richer** | **Richest** |
| **Afghanistan (2015)** | 6412 | 27·46 | 84·6 | 85·6 | 6·99 | 6·15 | 1·26 | 22 | 24·42 | 21·5 | 18·98 | 13·09 |
| **Angola (2016)** | 1364 | 25·73 | 38·84 | 25·36 | 37·71 | 33·43 | 3·5 | 22·33 | 22·37 | 21·98 | 19·76 | 13·56 |
| **Armenia (2016)** | 174 | 26·4 | 45·25 | 0 | 3·56 | 37·48 | 58·96 | 13·34 | 19·99 | 22·11 | 20·09 | 24·48 |
| **Benin (2012)** | 1556 | 27.44 | 63·61 | 69·71 | 17·2 | 11·75 | 1·34 | 17·91 | 22·72 | 22 | 20·15 | 17.22 |
| **Burkina Faso (2010)** | 1730 | 26·99 | 82·03 | 81·93 | 11·85 | 5·6 | 0·46 | 18·76 | 22·74 | 21·78 | 20·06 | 16·65 |
| **Burundi (2016)** | 1420 | 28·42 | 88·94 | 43·19 | 44·35 | 11·3 | 1·16 | 21·74 | 19·32 | 20·85 | 19·35 | 18·73 |
| **Cambodia (2014)** | 934 | 25·53 | 82·8 | 10·76 | 47·11 | 38·07 | 4·06 | 17·44 | 22·57 | 17·9 | 20·28 | 21·8 |
| **Cameroon (2011)** | 1512 | 26·38 | 56·29 | 27·49 | 37·93 | 31·58 | 3 | 22·54 | 22·79 | 17·97 | 20·37 | 16·33 |
| **Comoros (2012)** | 351 | 27·14 | 75·71 | 35·32 | 31·14 | 29·28 | 4·13 | 16·97 | 25·56 | 23·02 | 15·43 | 19·02 |
| **Congo (2012)** | 1031 | 26·6 | 35·45 | 7·03 | 27·69 | 60·73 | 4·54 | 20·09 | 23·45 | 21·42 | 20·06 | 14·98 |
| **Cote d'Ivoire (2012)** | 1032 | 26·3 | 58·27 | 59·28 | 24·94 | 13·54 | 2·24 | 21·22 | 23·51 | 19·6 | 19·07 | 16·58 |
| **Dominican Republic (2013)** | 479 | 24·19 | 23·34 | 2·56 | 34·26 | 40·4 | 22·78 | 23·57 | 23·96 | 25·55 | 16·29 | 10·63 |
| **Ethiopia (2016)** | 1135 | 27·23 | 85·83 | 53·2 | 35·03 | 8·28 | 3·49 | 22·67 | 23·33 | 18·77 | 17·45 | 17·78 |
| **Gabon (2012)** | 814 | 26·28 | 12·65 | 5·42 | 21·41 | 63·01 | 10·16 | 18·29 | 22·98 | 19·06 | 21·29 | 18·38 |
| **Gambia (2013)** | 830 | 26·85 | 54·38 | 58·51 | 18·38 | 20·31 | 2·8 | 20·84 | 20·95 | 24·1 | 16·05 | 18·07 |
| **Ghana (2014)** | 663 | 28·81 | 50·32 | 23·78 | 16·62 | 52·71 | 6·89 | 17·97 | 18·69 | 20·56 | 18·14 | 24·64 |
| **Guatemala (2015)** | 1427 | 25·04 | 63·98 | 13·29 | 54·99 | 27·86 | 3·86 | 24·83 | 22·74 | 20·35 | 17·07 | 15·02 |
| **Haiti (2012)** | 837 | 26·55 | 62·38 | 17·49 | 42·89 | 35·59 | 4·03 | 18·74 | 20·25 | 25·36 | 22·43 | 13·23 |
| **Honduras (2012)** | 1214 | 24·62 | 52·25 | 4·23 | 55·51 | 35·2 | 5·05 | 21·43 | 20·22 | 21·44 | 21·04 | 15·87 |
| **India (2016)** | 31123 | 24·27 | 71·5 | 24·5 | 12·6 | 49·3 | 13·6 | 23·2 | 21·6 | 20·4 | 18·2 | 16·6 |
| **Indonesia (2012)** | 1950 | 27·86 | 49·97 | 1 | 27·89 | 56·93 | 14·18 | 20·81 | 18·05 | 21·73 | 20·7 | 18·71 |
| **Kenya (2014)** | 1944 | 26·3 | 60·54 | 12·32 | 50·83 | 25·85 | 11·01 | 23·46 | 18·27 | 17·83 | 19·14 | 21·3 |
| **Kyrgyz Republic (2012)** | 551 | 25·81 | 66·23 | 0 | 1·06 | 55·83 | 43·11 | 22·6 | 18·09 | 21·09 | 17·81 | 20·86 |
| **Lesotho (2014)** | 284 | 25·18 | 69·13 | 1·65 | 42·54 | 48·63 | 7·19 | 17·26 | 17·53 | 22·76 | 24·21 | 18·24 |
| **Liberia (2013)** | 765 | 26·29 | 48·44 | 37·19 | 35·61 | 24·86 | 2·35 | 21·32 | 22·72 | 22·52 | 15·67 | 17·76 |
| **Malawi (2016)** | 1874 | 24·92 | 85·34 | 9·48 | 65·02 | 22·63 | 2·88 | 22·98 | 23·51 | 18·6 | 17·51 | 17·41 |
| **Mali (2013)** | 1202 | 26·98 | 84·47 | 79·58 | 10·06 | 9·75 | 0·62 | 21·42 | 19·57 | 22·65 | 20·17 | 16·2 |
| **Mozambique (2011)** | 1516 | 26·02 | 74·25 | 35·46 | 52·81 | 11 | 0·73 | 25·62 | 23·65 | 18·23 | 19·03 | 13·48 |
| **Myanmar (2016)** | 466 | 28·55 | 77·05 | 13·59 | 43·6 | 33·98 | 8·84 | 29·84 | 20·02 | 16·34 | 16·17 | 17·64 |
| **Namibia (2013)** | 600 | 26·64 | 41·85 | 7·19 | 19·1 | 63·21 | 10·5 | 16·26 | 19·69 | 20 | 25 | 19·06 |
| **Nepal (2016)** | 535 | 23·57 | 43·46 | 25·06 | 19·23 | 39·72 | 16 | 18·65 | 19·94 | 24·16 | 25·06 | 12·09 |
| **Niger (2012)** | 1591 | 27·29 | 86·51 | 83·25 | 11·5 | 4·93 | 0·28 | 17·34 | 19·94 | 22·7 | 20·07 | 19·94 |
| **Pakistan (2012)** | 1461 | 26.65 | 73.07 | 54·69 | 15·33 | 20·22 | 9·76 | 24·89 | 22·3 | 17·48 | 19·75 | 15·58 |
| **Philippines (2013)** | 686 | 26·75 | 52·97 | 0·93 | 18·3 | 53·24 | 27·54 | 26·13 | 22·25 | 18·92 | 18·95 | 13·74 |
| **Rwanda (2015)** | 984 | 28·46 | 82·35 | 11·15 | 70·3 | 15·36 | 3·19 | 20·14 | 20·61 | 21·07 | 18·91 | 19·27 |
| **Sierra Leone (2013)** | 1429 | 26·93 | 75·82 | 63·57 | 14·95 | 19·94 | 1·54 | 23·55 | 22·8 | 20·42 | 18·97 | 14·26 |
| **Tajikistan (2012)** | 734 | 24·59 | 79·37 | 2·11 | 5·57 | 80·66 | 11·66 | 16·51 | 23·96 | 20·78 | 22·75 | 16 |
| **Tanzania (2016)** | 1135 | 26·16 | 70·01 | 17·59 | 64·99 | 16·03 | 1·38 | 23·43 | 20·87 | 17·82 | 18·65 | 19·23 |
| **Timor-Leste (2016)** | 690 | 26·92 | 63·53 | 17·71 | 15·49 | 55·04 | 11·77 | 15·58 | 18·71 | 17·81 | 24·8 | 23·1 |
| **Togo (2014)** | 807 | 28·33 | 65·54 | 39·23 | 34·31 | 23·99 | 2·47 | 20·35 | 22·69 | 18·08 | 20·74 | 18·13 |
| **Uganda (2016)** | 1843 | 25·86 | 76·85 | 8·26 | 62·11 | 23·73 | 5·9 | 21·8 | 21·19 | 20·04 | 17·76 | 19·22 |
| **Zambia (2014)** | 1427 | 26·88 | 61·68 | 11·47 | 50·11 | 34·49 | 3·93 | 22·22 | 20·78 | 18·69 | 21·02 | 17·29 |

Supplementary Table 2: Characteristics of non-pregnant women across countries

| **Characteristics of non-pregnant women (n = 1230724)** | | | | | | | | | | | | |
| --- | --- | --- | --- | --- | --- | --- | --- | --- | --- | --- | --- | --- |
| **Country** | **Non-pregnant women (weighted)** | **Mean age** | **Rural dwellers (%)** | **Highest education level (%)** | | | | **Wealth Index (%)** | | | | |
|  |  |  |  | **No education** | **Primary** | **Secondary** | **Higher** | **Poorest** | **Poorer** | **Middle** | **Richer** | **Richest** |
| **Afghanistan (2015)** | 23049 | 32·19 | 74·48 | 82·93 | 8·16 | 6·84 | 2·06 | 19·49 | 19·24 | 19·56 | 20·8 | 20·9 |
| **Angola (2016)** | 13015 | 27·83 | 29·47 | 21·77 | 34·5 | 38·84 | 4·89 | 16·28 | 17·13 | 19·21 | 22·74 | 24·63 |
| **Armenia (2016)** | 5942 | 31·65 | 40·06 | 0·08 | 6·56 | 40·04 | 53·31 | 17·8 | 20·31 | 18·57 | 21·07 | 22·25 |
| **Benin (2012)** | 15043 | 29·05 | 52·5 | 58·46 | 17·54 | 22·03 | 1·96 | 16·73 | 17·71 | 18·55 | 21·57 | 25·45 |
| **Burkina Faso (2010)** | 15357 | 29·99 | 71·91 | 73·03 | 13·83 | 12·08 | 1·02 | 17·4 | 18·2 | 18·72 | 19·85 | 25·83 |
| **Burundi (2016)** | 15849 | 28·24 | 86·91 | 35·62 | 38·77 | 24·35 | 1·25 | 18·93 | 19·92 | 19·94 | 19·53 | 21·67 |
| **Cambodia (2014)** | 16644 | 30·57 | 81·43 | 12·92 | 47·11 | 35·34 | 4·64 | 17·91 | 18·64 | 19·31 | 20·57 | 23·58 |
| **Cameroon (2011)** | 13914 | 28·08 | 45·03 | 19·19 | 33·35 | 41·73 | 5·73 | 15·38 | 17·3 | 19·06 | 23·06 | 25·2 |
| **Comoros (2012)** | 4978 | 27·61 | 66·32 | 30·69 | 18·82 | 40·38 | 9·81 | 16·24 | 19·75 | 20·37 | 21·46 | 22·19 |
| **Congo (2012)** | 9788 | 28·8 | 30·97 | 5·63 | 23·4 | 65·7 | 5·27 | 16·37 | 19·17 | 20·58 | 21·89 | 21·99 |
| **Cote d'Ivoire (2012)** | 9028 | 28·61 | 47·51 | 52·5 | 25·41 | 19·29 | 2·8 | 17·17 | 16·57 | 18·01 | 20·96 | 27·29 |
| **Dominican Republic (2013)** | 8893 | 30·25 | 24·21 | 1·95 | 30·09 | 39·82 | 28·14 | 15·58 | 19·53 | 20·42 | 22·31 | 22·17 |
| **Ethiopia (2016)** | 14548 | 28·24 | 77·21 | 47·39 | 35·01 | 11·85 | 5·76 | 16·33 | 17·49 | 19·01 | 19·94 | 27·23 |
| **Gabon (2012)** | 7608 | 28·66 | 11·22 | 4·32 | 21·19 | 64·7 | 9·79 | 14·1 | 18·85 | 21·41 | 22·43 | 23·21 |
| **Gambia (2013)** | 9403 | 27·42 | 43·09 | 45·42 | 13·32 | 35·56 | 5·7 | 16·72 | 18·16 | 18·37 | 21·28 | 25·47 |
| **Ghana (2014)** | 8733 | 29·95 | 45·93 | 18·71 | 17·88 | 57·11 | 6·3 | 15·94 | 17·32 | 20·63 | 22·87 | 23·25 |
| **Guatemala (2015)** | 24487 | 28·91 | 54·18 | 14·3 | 45·59 | 33·52 | 6·59 | 16·91 | 18·54 | 19·89 | 22·18 | 22·48 |
| **Haiti (2012)** | 13450 | 28·26 | 51·89 | 14·64 | 35·86 | 43·8 | 5·7 | 15 | 15·92 | 19·31 | 22·84 | 26·93 |
| **Honduras (2012)** | 21543 | 29 | 45·45 | 3·91 | 49·83 | 38·19 | 8·06 | 15·57 | 17·72 | 20·82 | 22·94 | 22·95 |
| **India (2016)** | 668563 | 30·23 | 65·1 | 27·6 | 12·46 | 47·22 | 12·72 | 17·47 | 19·47 | 20·56 | 21·29 | 21·21 |
| **Indonesia (2012)** | 43657 | 31·61 | 47·73 | 3·39 | 33·4 | 51·13 | 12·08 | 16·86 | 19·31 | 20·2 | 21·39 | 22·23 |
| **Kenya (2014)** | 29135 | 29·05 | 59·08 | 6·65 | 50·24 | 31·92 | 11·19 | 15·04 | 17·51 | 19·51 | 21·2 | 26·73 |
| **Kyrgyz Republic (2012)** | 7657 | 29·95 | 62·33 | 0·05 | 0·38 | 56·15 | 43·42 | 17·46 | 17·94 | 18·57 | 20·49 | 25·55 |
| **Lesotho (2014)** | 6338 | 28·53 | 63·2 | 1·0 | 38·39 | 51·76 | 8·85 | 14·37 | 15·52 | 18·61 | 24·25 | 27·25 |
| **Liberia (2013)** | 8474 | 28·77 | 38·18 | 32·82 | 30·71 | 31·96 | 4·51 | 16·73 | 17·12 | 18·97 | 22·74 | 24·44 |
| **Malawi (2016)** | 22688 | 28·37 | 81·39 | 12·34 | 61·83 | 22·8 | 3·03 | 19·02 | 18·74 | 18·89 | 19·18 | 24·17 |
| **Mali (2013)** | 9222 | 28·83 | 74·01 | 75·33 | 9·15 | 14·13 | 1·39 | 18·38 | 18·61 | 18·43 | 20·49 | 24·09 |
| **Mozambique (2011)** | 12229 | 28·91 | 64·16 | 30·71 | 49·92 | 17·95 | 1·42 | 18·06 | 17·93 | 18·8 | 20·4 | 24·82 |
| **Myanmar (2016)** | 12419 | 31·79 | 70·52 | 12·42 | 41·09 | 36·15 | 10·34 | 17·19 | 18·64 | 20·59 | 21·15 | 22·43 |
| **Namibia (2013)** | 8576 | 29 | 43·55 | 4·38 | 19·63 | 65·88 | 10·11 | 15·52 | 17·57 | 19·53 | 22·93 | 24·45 |
| **Nepal (2016)** | 12327 | 29·57 | 36·97 | 33·64 | 16·61 | 34·91 | 14·84 | 16·84 | 19·62 | 20 | 21·35 | 22·2 |
| **Niger (2012)** | 9569 | 29·06 | 80·35 | 79·46 | 11·42 | 8·37 | 0·63 | 18·22 | 18·65 | 19·23 | 20·63 | 23·27 |
| **Pakistan (2012)** | 12097 | 33·21 | 65·76 | 57·35 | 15·97 | 17·44 | 9·24 | 18·4 | 19·43 | 20·21 | 20·67 | 21·3 |
| **Philippines (2013)** | 15469 | 30·17 | 46·59 | 1·17 | 15·95 | 48·81 | 34·06 | 15·78 | 17·67 | 19·84 | 22·25 | 24·46 |
| **Rwanda (2015)** | 12513 | 28·79 | 80·4 | 12·43 | 63·83 | 21·09 | 2·65 | 18·88 | 19·41 | 19·1 | 19·57 | 23·05 |
| **Sierra Leone (2013)** | 15229 | 28·55 | 63·31 | 55·06 | 13·9 | 27·9 | 3·14 | 18·08 | 17·86 | 18·7 | 20·47 | 24·89 |
| **Tajikistan (2012)** | 8922 | 29·23 | 74·65 | 2·01 | 3·71 | 81·04 | 13·23 | 19·7 | 19·47 | 19·63 | 20·22 | 20·98 |
| **Tanzania (2016)** | 12131 | 28·93 | 63·15 | 14·4 | 61·61 | 22·61 | 1·38 | 16·32 | 16·79 | 17·53 | 21·52 | 27·85 |
| **Timor-Leste (2016)** | 11917 | 28·84 | 67·02 | 21·98 | 15·23 | 51·87 | 10·92 | 16·59 | 18·11 | 19·3 | 21·82 | 24·18 |
| **Togo (2014)** | 8673 | 29·44 | 53·58 | 31·08 | 33·4 | 32·08 | 3·44 | 16·31 | 16·36 | 18·2 | 23 | 26·14 |
| **Uganda (2016)** | 16663 | 28·16 | 72·89 | 9·78 | 56·92 | 25·22 | 8·08 | 17·07 | 18·04 | 18·55 | 20·14 | 26·2 |
| **Zambia (2014)** | 14984 | 28·5 | 53·03 | 8·09 | 46·5 | 40·24 | 5·17 | 16·96 | 17.12 | 18·76 | 21·42 | 25·74 |

Supplementary Table 3: Tobacco use among pregnant and non-pregnant women in SEAR

| **Tobacco use among pregnant women** | | | | | |
| --- | --- | --- | --- | --- | --- |
| **Country** | **Response rate %** | **Response rate n (weighted)** | **Exclusive smokeless % (95% CI)** | **Exclusive smoking % (95% CI)** | **Dual % (95% CI)** |
| **India (2016)** | 100 | 31123 | 3·21 (2·94-3·5) | 0·43 (0·35-0·52) | 0·005 (0·002-0·11) |
| **Indonesia (2012)** | 99·96 | 1949 | 0·28 (0·12-0·65) | 0·73 (0·4-1·35) | 0 |
| **Myanmar (2016)** | 99·97 | 465 | 0 | 3·36 (1·95-5·73) | 0 |
| **Nepal (2016)** | 100 | 535 | 0·46 (0·13-1·63) | 1·66 (0·68-3·99) | 0 |
| **Timor-Leste (2016)** | 100 | 690 | 0 | 4·92 (2·85-8·37) | 0 |
| **Total** |  | **34762** | **0**·**45 (0**·**002 - 2**·**29)** | **1**·**81 (0**·**61 - 3**·**61)** | **0**·**01 (0**·**00 - 0**·**03)** |
| **Tobacco use among non-pregnant women** | | | | | |
| **Country** | **Response rate %** | **Response rate n (weighted)** | **Exclusive smokeless % (95% CI)** | **Exclusive smoking % (95% CI)** | **Dual % (95% CI)** |
| **India (2016)** | 100 | 668563 | 4·61 (4·48 - 4·73) | 0·76 (0·72 - 0·81) | 0·05 (0·04 - 0·06) |
| **Indonesia (2012)** | 99·9 | 43613 | 0·31 (0·23 - 0·42) | 2·37 (2·13 - 2·62) | 0·1 (0·06 - 0·17) |
| **Myanmar (2016)** | 100 | 12419 | 0·17 (0·09 -0·29) | 3·63 (3·14 - 4·19) | 0·03 (0·009 - 0·09 |
| **Nepal (2016)** | 100 | 12327 | 2·68 (2·32 - 3·09) | 5·29 (4·76 - 5·88) | 0·71 (0·51 - 0·99) |
| **Timor-Leste (2016)** | 100 | 11917 | 0·14 (0·08 - 0·25) | 3·97 (3·4 - 4·63) | 0·09 (0·04 - 0·17) |
| **Total** |  | **748839** | **1**·**06 (0**·**01 - 3**·**86)** | **2**·**97 (1**·**30 - 5**·**29)** | **0**·**14 (0**·**04 - 0**·**28)** |

*Supplementary Figure 1: Exclusive smokeless tobacco use among pregnant and non-pregnant women*

*Supplementary Figure 2: Exclusive smoking among pregnant and non-pregnant women*
